# A Brainstem‐Dominant Acute Disseminated Encephalomyelitis Presenting With Magnetic Resonance Imaging Findings Similar to Those of Chronic Lymphocytic Inflammation With Pontine Perivascular Enhancement Responsive to Steroid Syndrome

**DOI:** 10.1002/ccr3.70252

**Published:** 2025-02-26

**Authors:** Masahiko Ezoe, Risa Hirata, Masafumi Kosugi, Masaki Tago

**Affiliations:** ^1^ Department of General Medicine Saga University Hospital Saga Japan; ^2^ Department of General Medicine National Hospital Organization Ureshino Medical Center Saga Japan; ^3^ Neurology National Hospital Organization Ureshino Medical Center Saga Japan

**Keywords:** acute disseminated encephalomyelitis, general medicine, magnetic resonance imaging, neurology

## Abstract

Acute disseminated encephalomyelitis can present with brainstem symptoms and bilateral symmetrical lesions in the basal ganglia. In such cases, differentiation from chronic lymphocytic inflammation with pontine perivascular enhancement responsive to steroids syndrome is necessary.

A 78‐year‐old man, diagnosed with hypertension and chronic obstructive pulmonary disease, presented to our hospital with an acute fever. He was alert (Glasgow Coma Scale E4V5M6) with a body temperature of 39.0°C, a respiratory rate of 16 breaths/min, and O_2_ saturation of 93% on 4 L/min O_2_. Physical examination revealed only coarse crackles in both lower lung fields. Blood tests showed a white blood cell count, neutrophils, and C‐reactive protein levels of 8840 cells/μL, 87.4%, and 5.7 mg/dL (< 0.14 mg/dL), respectively. However, the chest computed tomography (CT) revealed bronchopneumonia in both lower lobes. Despite ceftriaxone administration (2 g/day), his fever and inflammatory response persisted. Aspiration occurred on Day 6, followed by lower limb weakness with manual muscle testing of 3 on Day 7. The patient became drowsy, and a head CT revealed a new hypoattenuation on the pons' right side. On Day 8, he exhibited progressive disturbances in consciousness and left eye abduction impairment, with a positive left Babinski sign. Head magnetic resonance imaging (MRI) showed symmetrical high signal intensities on FLAIR images in the basal ganglia, external capsule, and thalamus, with punctate high signals in the pons' median part and bilaterally (predominantly on the right side, Figure 1). The imaging findings matched the clinical symptoms and physical examination results. Cerebrospinal fluid (CSF) analysis revealed a mononuclear cell count and protein levels of 54 cells/μL and 67 mg/dL, respectively. These findings suggested a central inflammatory disease, including chronic lymphocytic inflammation with pontine perivascular enhancement responsive to steroids (CLIPPERS) syndrome; treatment with methylprednisolone (500 mg/day) was initiated for 3 days. Subsequently, the patient experienced a rapid defervescence, improved levels of consciousness, and resolution of dysphagia and left lower limb weakness. The clinical course and CSF analysis showing elevated myelin basic protein levels (157 pg/mL, reference value below 102.0 pg/mL) confirmed acute disseminated encephalomyelitis (ADEM). Antinuclear antibodies, myeloperoxidase‐antineutrophilic cytoplasmic antibody (ANCA), cytoplasmic‐ANCA, soluble interleukin‐2 receptor, angiotensin‐converting enzyme, and anti‐aquaporin 4 antibody antibodies were negative. The steroid treatment was gradually reduced and stopped on Day 9. Currently, at 16 months post discharge, he remains symptom‐free without any relapse, with improved head MRI findings (Figure 2).

**FIGURE 1:**
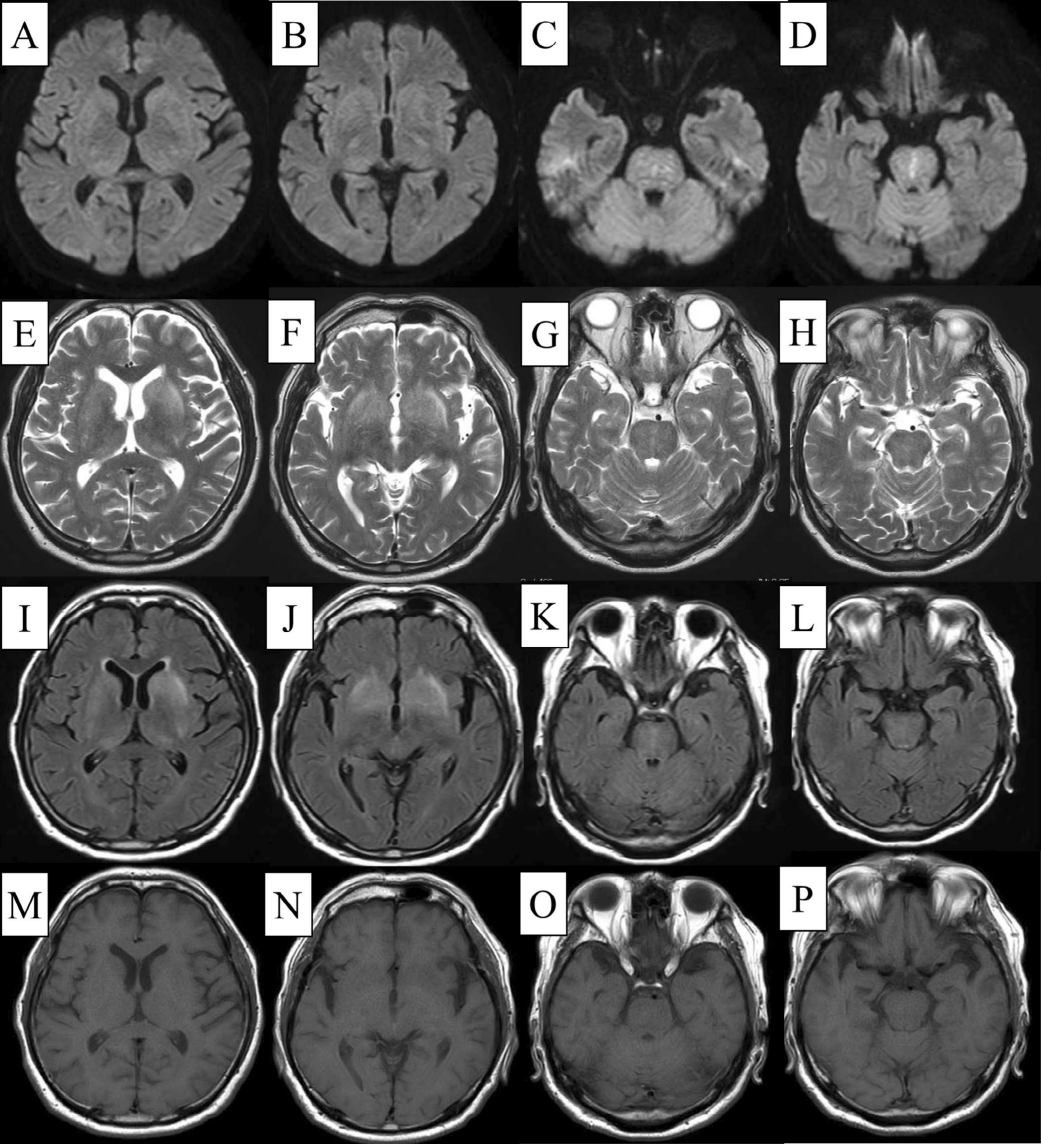
Head magnetic resonance image (horizontal section; A–D are DWI, E–H are T2WI, I–L are FLAIR, and M–P are T1WI). DWI, T2WI, and FLAIR images reveal symmetrical high signal intensities in the basal ganglia (A, E, I), external capsule, thalamus (B, F, J), and median part of the pons (C, G, K), with a predominance on the right side (D, H, L). No signal changes occurred on T1WI (M, N, O, P).

**FIGURE 2:**
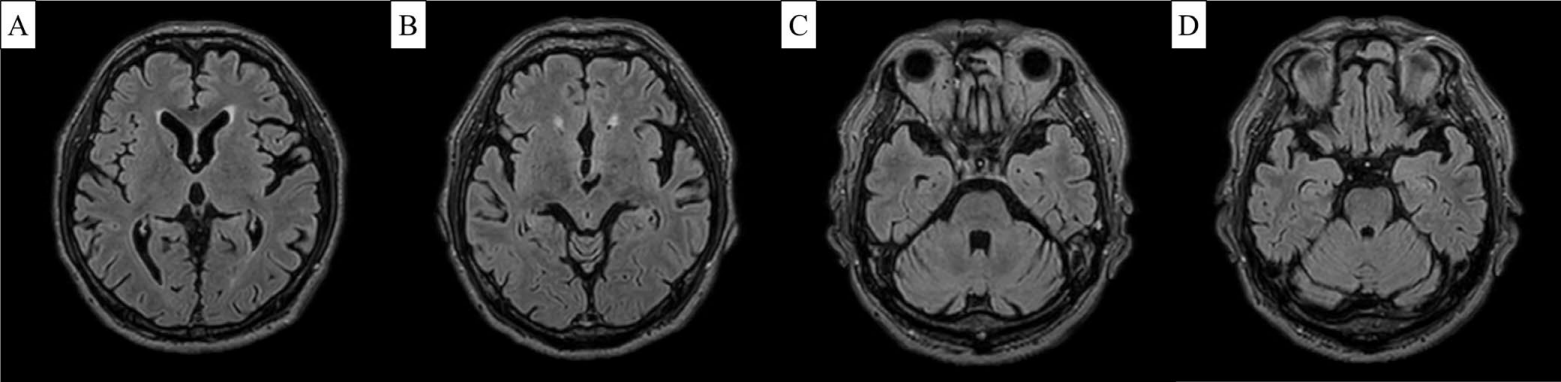
Head magnetic resonance image 16 months after onset (horizontal section, FLAIR images). FLAIR images show that the high signal regions in the bilateral basal ganglia (A), external capsule, thalamus (B), and pons (C, D) have disappeared.

Multiple, bilateral, asymmetric cerebellar lesions on head MRI characterize ADEM [1]. Differentiating ADEM from neurosarcoidosis, multiple sclerosis, and CLIPPERS syndrome is crucial. Here, brainstem symptoms were predominant, and the MRI showed symmetrical lesions in the basal ganglia and salt‐and‐pepper‐like findings in the brainstem characteristic of CLIPPERS syndrome [2], which posed a diagnostic challenge. Although elevated CSF myelin basic protein sensitivity and specificity have not been assessed in ADEM [3], its elevation is frequently observed in ADEM. Conversely, no such elevation has been reported in CLIPPERS syndrome. While most CLIPPERS syndrome cases relapse after steroid cessation [2], relapses are less common in ADEM. ADEM can present with bilateral lesions in the deep gray matter and brainstem [1]; thus, here, the ADEM diagnosis was consistent with the literature.

In conclusion, ADEM can present with brainstem symptoms and bilateral symmetrical lesions in the basal ganglia, requiring differentiation from CLIPPERS syndrome; therefore, measuring CSF myelin basic protein may be helpful.

## Author Contributions

**Masahiko Ezoe:** conceptualization, investigation, writing – original draft. **Risa Hirata:** conceptualization, investigation, writing – original draft. **Masafumi Kosugi:** investigation, writing – review and editing. **Masaki Tago:** conceptualization, writing – review and editing.

## Ethics Statement

This manuscript complies with the provisions of the 1995 Declaration of Helsinki (as revised in Brazil 2013).

## Consent

Written informed consent was obtained from the patient to publish this report in accordance with the journal's patient consent policy.

## Conflicts of Interest

The authors declare no conflicts of interest.

## Data Availability

The data supporting this study's findings are available from the corresponding author upon reasonable request.

## References

[1] K. Li , M. Li , L. Wen , Q. Wang , X. Ding , and J. Wang , “Clinical Presentation and Outcomes of Acute Disseminated Encephalomyelitis in Adults Worldwide: Systematic Review and Meta‐Analysis,” Frontiers in Immunology 13 (2022): 870867, 10.3389/fimmu.2022.870867. 35757742 PMC9218070

[2] M. Al‐Chalabi , N. R. DelCimmuto , A. Beran , et al., “Clinical Characteristics, Management, and Outcomes of CLIPPERS: A Comprehensive Systematic Review of 140 Patients From 100 Studies,” Multiple Sclerosis and Related Disorders 68 (2022): 104112, 10.1016/j.msard.2022.104112. 36029706

[3] A. C. Anilkumar , L. A. Foris , and P. Tadi , “Acute Disseminated Encephalomyelitis,” in StatPearls (StatPearls Publishing, 2024). 28613684

